# Restoration of motor function following spinal cord injury via optimal control of intraspinal microstimulation: toward a next generation closed-loop neural prosthesis

**DOI:** 10.3389/fnins.2014.00296

**Published:** 2014-09-17

**Authors:** Peter J. Grahn, Grant W. Mallory, B. Michael Berry, Jan T. Hachmann, Darlene A. Lobel, J. Luis Lujan

**Affiliations:** ^1^Mayo Clinic College of Medicine, Mayo ClinicRochester, MN, USA; ^2^Department of Neurologic Surgery, Mayo ClinicRochester, MN, USA; ^3^Department of Neurosurgery, Cleveland ClinicCleveland, OH, USA; ^4^Department of Physiology and Biomedical Engineering, Mayo ClinicRochester, MN, USA

**Keywords:** spinal cord injury, brain machine interface, closed-loop control, feedback control, neuroprosthetics, sensors, implantable systems

## Abstract

Movement is planned and coordinated by the brain and carried out by contracting muscles acting on specific joints. Motor commands initiated in the brain travel through descending pathways in the spinal cord to effector motor neurons before reaching target muscles. Damage to these pathways by spinal cord injury (SCI) can result in paralysis below the injury level. However, the planning and coordination centers of the brain, as well as peripheral nerves and the muscles that they act upon, remain functional. Neuroprosthetic devices can restore motor function following SCI by direct electrical stimulation of the neuromuscular system. Unfortunately, conventional neuroprosthetic techniques are limited by a myriad of factors that include, but are not limited to, a lack of characterization of non-linear input/output system dynamics, mechanical coupling, limited number of degrees of freedom, high power consumption, large device size, and rapid onset of muscle fatigue. Wireless multi-channel closed-loop neuroprostheses that integrate command signals from the brain with sensor-based feedback from the environment and the system's state offer the possibility of increasing device performance, ultimately improving quality of life for people with SCI. In this manuscript, we review neuroprosthetic technology for improving functional restoration following SCI and describe brain-machine interfaces suitable for control of neuroprosthetic systems with multiple degrees of freedom. Additionally, we discuss novel stimulation paradigms that can improve synergy with higher planning centers and improve fatigue-resistant activation of paralyzed muscles. In the near future, integration of these technologies will provide SCI survivors with versatile closed-loop neuroprosthetic systems for restoring function to paralyzed muscles.

## Introduction

Approximately 300,000 individuals in the United States, and more than 2.5 million individuals worldwide, are affected by traumatic spinal cord injury (SCI) (National Spinal Cord Injury Statistical Center, 2013). Overall health-care related cumulative costs are estimated to exceed $9 billion annually in the United States alone (DeVivo, 2012). In 2010, 36.5% of SCI resulted from motor vehicle accidents, 28.5% from falls, 14% from violence (including gunshot wounds), 9% from sports accidents, and 11% from other incidences not reported in detail (National Spinal Cord Injury Statistical Center, 2013). The demographic profile has changed over the last 40 years to involve older aged individuals. However, males still comprise the majority of injuries (Sekhon and Fehlings, 2001; DeVivo, 2012; Lenehan et al., 2012; National Spinal Cord Injury Statistical Center, 2013).

Traumatic SCI can occur when an excessive load to the spinal column is transmitted (directly or indirectly) to the spinal cord (Rowland, 1991; Watson et al., 2009). Damage to the spinal cord begins at the moment of injury, when displaced fragments of bone, disc material, or ligaments typically cause bruises or tears to spinal cord tissue (McDonald and Sadowsky, 2002). However, paralysis has been observed with no radiographic evidence of damage to the spinal cord or vertebral column (Pang and Wilberger, 1982; Mirovsky et al., 2005; Mahajan et al., 2013). Regardless of the injury mechanism, SCI involves permanent sensorimotor and autonomic deficits (Scivoletto et al., 2014), with long term complications including muscle atrophy and increased risk of cardiovascular disease (Phillips et al., 1998; Chen et al., 1999).

Most spinal cord injuries do not completely sever the spinal cord (Marino et al., 2003; National Institute of Neurological Disorders and Stroke, 2013). Instead, key pathways necessary for signal transmission between the brain and the rest of the body are disrupted. Spinal cord injuries can be classified as complete and incomplete injuries (Marino et al., 2003). Complete injuries are indicated by a total lack of sensory and motor function below the level of injury. In contrast, the ability to convey messages to or from the brain is not completely lost in cases of incomplete injury. That is, limited sensation and movement remain below the level of injury. Although SCI interrupts connections between the brain and effector muscles, key planning, coordination, and effector centers above and below the injury remain intact (Krajl et al., 1986; Triolo et al., 1996; Jilge et al., 2004; Minassian et al., 2004; Fisher et al., 2008, 2009; Yanagisawa et al., 2011; Wang et al., 2013; Collinger et al., 2014). Functional electrical stimulation (FES) is a form of therapy that applies external currents into intact neuromuscular circuitry below the level of injury, activating intact neural components to cause muscle contractions that can lead to restoration of motor function (Jackson and Zimmermann, 2012).

**Figure 1:**
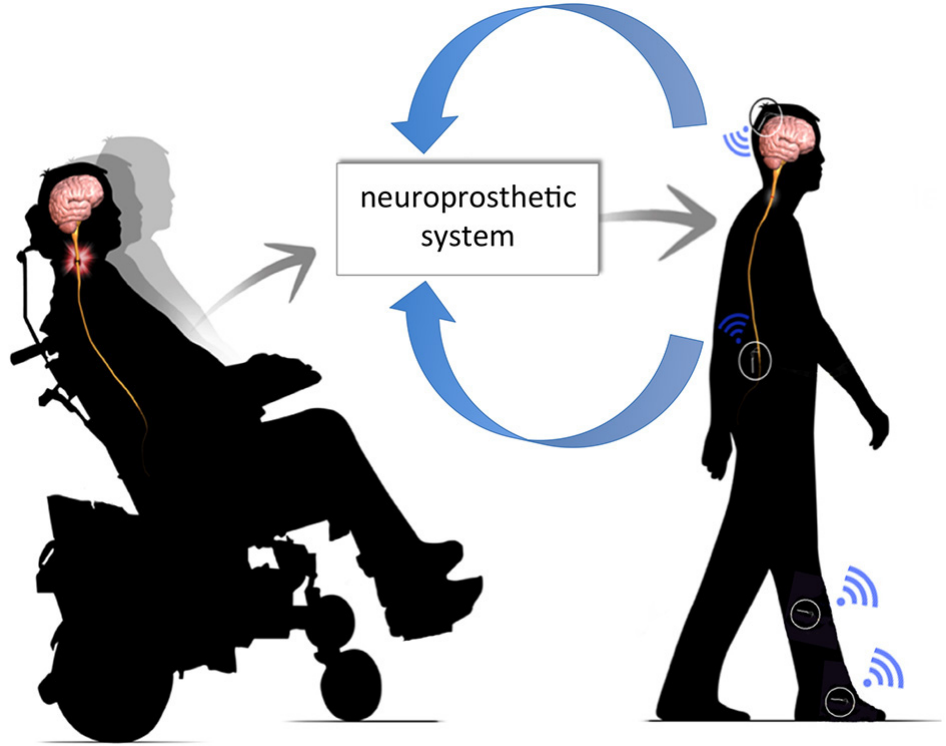
**Neuroprosthetic system**. The neuroprosthetic system is capable of interpreting volitional movement signals from the brain, integrating these commands with sensor feedback (e.g., joint angle, limb velocity, etc.) and, delivering appropriate commands into intact neural circuitry below the level of injury.

This manuscript reviews current therapeutic applications of electrical stimulation of the spine for providing functional coordination of muscle contraction and restoring function to paralyzed muscles. Additionally, this manuscript describes the development of neurostimulation technologies and control strategies, combining brain signals, optimal control algorithms, and emerging FES strategies to develop a clinically-translatable FES system that optimizes restoration of neurologic function following SCI (Figure 1).

## Electrical stimulation of excitable tissue

The use of electrical stimulation for investigating the function of the nervous system began with the Italian physician and scientist Luigi Galvani (Galvani and Aldini, 1792). Galvani discovered that nerves and muscles are electrically excitable, and was able to evoke muscle contractions in frog legs by stimulating them with brief jolts of electricity, produced by static generators (Hambrecht, 1992). Since then, it has been well established that nerve cells can be activated using electrical currents delivered into neural tissue via stimulating electrodes (Glenn et al., 1976; Branner et al., 2001; Brill et al., 2009; Kilgore et al., 2009; Kent and Grill, 2013; Nishimura et al., 2013). Active nerve cells fire electrical impulses, also known as action potentials, that travel along the nerve axon and propagate across neuromuscular junctions via neurotransmitter signaling (Bean, 2007; Meriney and Dittrich, 2013). In turn, this signaling mechanism causes muscle fibers connected to nerve fibers (i.e., motor unit) to contract (Hughes et al., 2006).

## Electrically evoked muscle activation

The strength of stimulation-evoked muscle contractions can be controlled by varying the frequency, amplitude, and pulsewidth of the external stimuli (Grobelnik, 1973; Kralj et al., 1988; Kralj and Bajd, 1989; Bhadra and Peckham, 1997). At low frequencies, individual muscle twitches are evoked with each stimulus pulse. At higher frequencies, responses to individual stimuli fuse and muscles respond with smooth contractions. Higher stimulus frequencies produce stronger muscle contractions, but also increase the rate of muscle fatigue (Tanae et al., 1973; McDonnall et al., 2004; Bamford, 2005). Activation of motor units can be achieved using different stimulation modalities: transcutaneous stimulation, percutaneous stimulation, intramuscular stimulation, peripheral nerve stimulation, and spinal stimulation.

### Transcutaneous stimulation

Transcutaneous stimulation, also known as surface stimulation, relies on stimulating electrodes placed on the skin surface directly over the muscle motor points (i.e., locations that produce an optimal balance between contraction strength and stimulation amplitude) (Hirokawa et al., 1990; Scremin et al., 1999; Mangold et al., 2005). This non-invasive, reversible, and inexpensive technique has been successfully used in locomotion and hand grasp systems (Kralj and Bajd, 1989; Popovic et al., 2005). However, transcutaneous muscle stimulation has multiple practical limitations. Specifically, the skin offers a high resistance compared to muscle tissue (Bîrlea et al., 2014). For this reason, higher stimulation currents (>30 mA) are required to achieve desired motor responses using surface stimulation (Triolo et al., 2001; Lujan and Crago, 2009). Additionally, the limited degree of selectivity can lead to activation of antagonist muscle groups or an inability to selectively activate deep muscle groups (Schmit and Mortimer, 1997; Triolo et al., 2001). Furthermore, current spread due to suboptimal electrode placement and limited stimulation specificity can result in pain (Niddam et al., 2001).

### Percutaneous stimulation

Percutaneous stimulation systems rely on intramuscular needle electrodes that pass through the skin and stimulate target muscles (Caldwell and Reswick, 1975; Stanic et al., 1978; Malezic et al., 1984; Marsolais and Kobetic, 1986; Bogataj et al., 1989). This allows activation of deep muscles and provides isolated, selective, and repeatable muscle contractions. Percutaneous stimulation requires lower stimulation intensities compared to transcutaneous stimulation. However, increased risks of infection, lead breakage, and movement restrictions limit the use of percutaneous stimulation outside of a laboratory environment (Knutson et al., 2002).

### Implanted intramuscular and peripheral nerve stimulation

Implanted neurostimulation systems are associated with electrical current delivery via both intramuscular and nerve cuff electrodes (Rabischong and Ohanna, 1992; Peckham et al., 2002; Guiraud et al., 2006). As the name implies, intramuscular stimulation relies on electrodes implanted directly into the muscle (Crago et al., 1980; Hobby et al., 2001; Peckham et al., 2001, 2002; Peckham and Knutson, 2005; Kilgore et al., 2008). Peripheral nerve stimulation relies on electrode cuffs that are surgically placed around nerves innervating target muscles (Stein et al., 1975; Hoffer et al., 1996; Strange and Hoffer, 1999; Sinkjaer, 2000; Branner et al., 2001; Brill et al., 2009; Fisher et al., 2009; Polasek et al., 2009). Although capable of evoking strong, selective, and repeatable muscle activation, intramuscular and nerve cuff stimulation techniques often recruit the largest and most fatigable motor units first, resulting in early fatigue onset (Popovic et al., 2002). Discontinuous activation of muscle compartments and interleaved frequency stimulation have both been reported to delay fatigue onset (Boom et al., 1993; McDonnall et al., 2004). Saigal et al. demonstrated fatigue-resistant stepping in a spinalized cat by stimulating the lumbrosacral cord via interleaved stimulation (Saigal et al., 2004). Interleaved stimulation reduces muscle fatigue by decreasing the stimulation frequency (Mushahwar and Horch, 1997; Tai et al., 2000). The asynchronous nature of interleaved stimulation is designed to evoke fused contractions despite a lack of tetanic firing in individual motor units. However, the limited number of controllable degrees of freedom, high power consumption, and other technological and practical limitations have restricted the widespread application of electrical stimulation therapy outside research environments (Peckham and Knutson, 2005; Ragnarsson, 2008; Creasey and Craggs, 2012).

### Spinal cord stimulation

Direct stimulation of the spinal cord may be advantageous over conventional FES techniques as spinal stimulation provides an opportunity to directly activate higher level circuitry, which oversees and coordinates motor function (Minassian et al., 2004, 2007; Bamford, 2005; Gerasimenko et al., 2008; Bamford and Mushahwar, 2011; Holinski et al., 2011; van den Brand et al., 2012; Angeli et al., 2014). Two modalities of spinal stimulation have been described: epidural and intraspinal stimulation.

In epidural stimulation, stimulating electrodes are placed directly over the spinal cord (Lavrov et al., 2008; Hachmann et al., 2013). Two recent studies reported that neuromodulation of spinal circuitry via epidural stimulation, combined with intense physical rehabilitation, was capable of allowing individuals with incomplete and complete SCI to process conceptual, auditory and visual feedback to regain voluntary control of paralyzed muscles for short durations of time. Results of these studies suggest some degree of residual connectivity through the area of SCI (Harkema et al., 2011; Angeli et al., 2014). These studies, although promising, require using rigorous patient selection and replication in larger patient populations.

In intraspinal microstimulation (ISMS), stimulating electrodes are implanted within the ventral gray matter of the spinal cord (Bamford and Mushahwar, 2011). ISMS is hypothesized to directly activate alpha motor neurons, preferentially activating fatigue resistant muscle fibers (Gorman, 2000; Bamford, 2005). Several studies have highlighted the potential of ISMS to restore bladder and respiratory function, as well as upper and lower extremity function in animal models (Mushahwar and Horch, 2000a,b; Mushahwar et al., 2002; Moritz et al., 2007; Bamford et al., 2010; Bamford and Mushahwar, 2011; Nishimura et al., 2013; Sunshine et al., 2013).

## Intraspinal microstimulation (ISMS)

Intraspinal stimulation has been extensively used to study the effects of electrical stimulation on the central nervous system, as well as synaptic delays and network interconnections across spinal pathways (Renshaw, 1946; Jankowska and Roberts, 1972a,b; Gustafsson and Jankowska, 1976). More recently, ISMS has been used to investigate the organization of motor circuitry within the spinal cord in amphibious, rodent, and feline animal models (Bizzi et al., 1991; Giszter et al., 1993; Tresch and Bizzi, 1999; Lemay et al., 2001, 2009; Saltiel et al., 2001; Lemay and Grill, 2004).

Similarly, over the past 15 years, ISMS has been used to investigate restoration of motor function in spinalized and anesthetized rodents and cats (Mushahwar et al., 2002; Bamford, 2005; Pikov et al., 2007; Yakovenko et al., 2007; Holinski et al., 2011; Kasten et al., 2013; Sunshine et al., 2013). Work performed by Lau et al. demonstrated that ISMS is capable of producing standing in cats for over 20 min (Lau et al., 2007). The lower stimulation amplitudes associated with intraspinal stimulation (in the order of a few microamperes) are believed to be, at least in part, responsible for the longer periods of muscle contraction observed (Bamford, 2005). Other studies suggest that the fatigue resistance observed with ISMS techniques is the result of preferential activation of type I slow-twitch fatigue-resistant motor fibers (Mushahwar, 2000; Mushahwar and Horch, 2000a; Saigal et al., 2004; Bamford, 2005; Nishimura et al., 2013). Moreover, Bamford et al. showed ISMS recruitment of up to 44% fatigue-resistant muscle fibers compared to less than 1% fatigue-resistant muscle fibers recruited using peripheral nerve cuff stimulation (Caldwell and Reswick, 1975; Marsolais and Kobetic, 1986; Bamford, 2005). As such, when combined with interleaved stimulation, ISMS has been associated with further decrease in muscle fatigue (Rack and Westbury, 1969; McDonnall et al., 2004; Lau et al., 2007; Mushahwar et al., 2007).

The close proximity of spinal motor centers to higher control centers responsible for controlling motor function, together with the improved fatigue response, make ISMS an excellent alternative for restoring locomotor function in individuals with SCI (Etlin et al., 2014; Guertin, 2014). However, before spinal or other electrical stimulation technology can be clinically used to optimally improve quality of life for individuals with SCI, appropriate stimulation control paradigms must be established.

## Optimal control paradigms

Electrical stimulation systems have been previously used to assist respiratory function (Kaneyuki et al., 1977; Gorman, 2000; Posluszny et al., 2014), hand grasp (Avestruz et al., 2008; Skarpaas and Morrell, 2009; Rosin et al., 2011; Gan et al., 2012; Basu et al., 2013; Grant and Lowery, 2013), locomotion (Behrend et al., 2009), as well as bladder and bowel function (Lee et al., 2004; Shon et al., 2010a,b; MacDonald et al., 2013) in patients with SCI. These FES systems have relied on a variety of control strategies, ranging from linear models to adaptive controllers, but all aimed at enhancing stimulation-evoked functional responses. Many neuroprosthetic control systems rely on feedforward configurations (Moro et al., 1999; Molinuevo et al., 2000), in which controller output depends only on user inputs (e.g., stimulus parameters). These controllers have fast response times, but do not make corrections if the target and actual outputs differ (Lee et al., 2009). Furthermore, these controllers will not alter their response in the face of unexpected internal or external perturbations (Blaha and Phillips, 1996; Lee et al., 2006). However, the highly non-linear nature of muscle responses, coupled with environmental perturbations found in activities of daily living, require that optimal neuroprosthetic control paradigms rely on feedback signals. Feedback-based control systems continuously monitor musculoskeletal system outputs and adjust stimulation parameters if the stimulation-evoked musculoskeletal system outputs (e.g., limb position, force) differ from the desired outputs (Lujan and Crago, 2009; Griessenauer et al., 2010; Chang et al., 2012). This guarantees the system can respond to and compensate for unforeseen perturbations. Feedback control has been previously used for control of hand grasp (Lujan and Crago, 2009), standing posture (Fraix et al., 2006; Rosin et al., 2011), and locomotion (Roham et al., 2007; Takmakov et al., 2010; Fitzgerald, 2014) in SCI individuals. Simple feedback control can be improved by using adaptive systems (Karniel and Inbar, 2000; Kobravi and Erfanian, 2012). Adaptive algorithms modify controller behavior in response to changes in the system and the environment (Chizek et al., 1988; Narendra, 1990; Narendra and Parthasarathy, 1990; Teixeira et al., 1991; Kostov et al., 1995; Davoodi and Andrews, 1998, 1999; Jonić et al., 1999; Abbas and Riener, 2001).

Studies have demonstrated the ability of neural networks to successfully control motor neuroprostheses, both in paraplegic (Riess and Abbas, 1999, 2000, 2001; Nataraj et al., 2013) and tetraplegic individuals (Fujita et al., 1998; Lujan and Crago, 2009). Artificial neural networks (ANNs) can model static and dynamic non-linear systems (Durfee, 1989; Funahashi, 1989; Hornik et al., 1989; Chakraborty et al., 1992; Barron, 1993; Lan et al., 1994; Piche, 1994; Graupe and Kordylewski, 1995; Hassoun, 1995; Kostov et al., 1995; Chang et al., 1996; Chen et al., 1997; Demuth and Beale, 2000). Additionally, ANNs can generalize from experimental input/output data, eliminating the need for analytical models of the system (Funahashi, 1989; Hornik et al., 1989; Graupe and Kordylewski, 1995; Hassoun, 1995; Narendra, 1996; Demuth and Beale, 2000). Furthermore, ANNs are less sensitive to noise and easily implemented in hardware (Narendra, 1996). Moreover, ANN-based controllers allow changes to the controller without requiring changes in data collection or controller training methods. Backpropagation neural networks have been used to model the non-linear relationship between stimulus intensity and stimulation-evoked responses (Fujita et al., 1998; Lujan and Crago, 2009). Additionally, ANNs have been successfully used to create inverse dynamic models of musculoskeletal systems for neuroprosthetic control (Chang et al., 1997; Yoshida et al., 2002). These models are particularly useful for learning the characteristics of electrically-activated muscles in coupled multi-joint systems acted upon by redundant muscles (Adamczyk and Crago, 1997, 2000; Lujan and Crago, 2009).

Thus, optimal neuroprosthetic control systems should rely on a combination of non-linear feedforward and feedback techniques in order to pre-emptively reduce the amount of error in real-time while minimizing time delays inherent to feedback control systems. Development of such optimal closed-loop neuroprosthetic controllers will require high-quality sensors that can withstand daily use under a wide range of daily life activities.

## Feedback signals for optimal control of neural prostheses

Neuroprosthetic systems with feedback control are capable of identifying, decoding, and extracting features from appropriate input signals in order to respond to unforeseen perturbations and changes in the environment (Bhadra et al., 2002; Dominici et al., 2012; Holinski et al., 2013). However, optimal feedback modulation for clinical application will require fully implantable smart sensors that provide consistent and reliable chronic information to the control system (Shih et al., 2012; Peckham and Kilgore, 2013). There is already a wide range of sensors that can detect and measure information about the system and its environment. The most commonly used sensors include electrophysiological sensors, chemical sensors, force transducers, and magnetic sensors. Electrophysiological sensors measure potential differences generated by muscle (i.e., myoelectric signals) and neural tissue (e.g., electroencephalogram, electrocorticogram, electroneurogram) (Leuthardt et al., 2004; Müller-Putz et al., 2005; Holinski et al., 2013). These sensors can monitor muscle state and evaluate expected muscle responses. In turn, this allows adaptation of stimulation parameters in the presence of muscle fatigue (Hayashibe et al., 2011; Zhang et al., 2013). Chemical sensors (e.g., carbon fiber microelectrodes coupled to fast scan cyclic voltammetry devices) can detect changes in stimulation-evoked analytes (e.g., neurotransmitters) (Bledsoe et al., 2009; Chang et al., 2012) that can be used to modulate stimulation levels. Force transducers (e.g., piezoelectric devices, accelerometers) can be used to detect changes in limb position, ground reaction forces, heel strike, and other events that are critical for event detection and optimal control of stimulation (Tan et al., 2004). Magnetic sensors detect changes in magnetic fields and can be used to detect limb position and orientation (Bhadra et al., 2002; Tan et al., 2004). However, having reliable sensors is not enough to develop an optimal feedback controller. In order for the signals measured by these sensors to be of clinical use, they must be properly decoded and integrated with both existing and novel neuroprosthetic control systems (Shadmehr et al., 2010). This will most likely happen in the way of a brain machine interface.

## Brain machine interfaces

**Figure 2:**
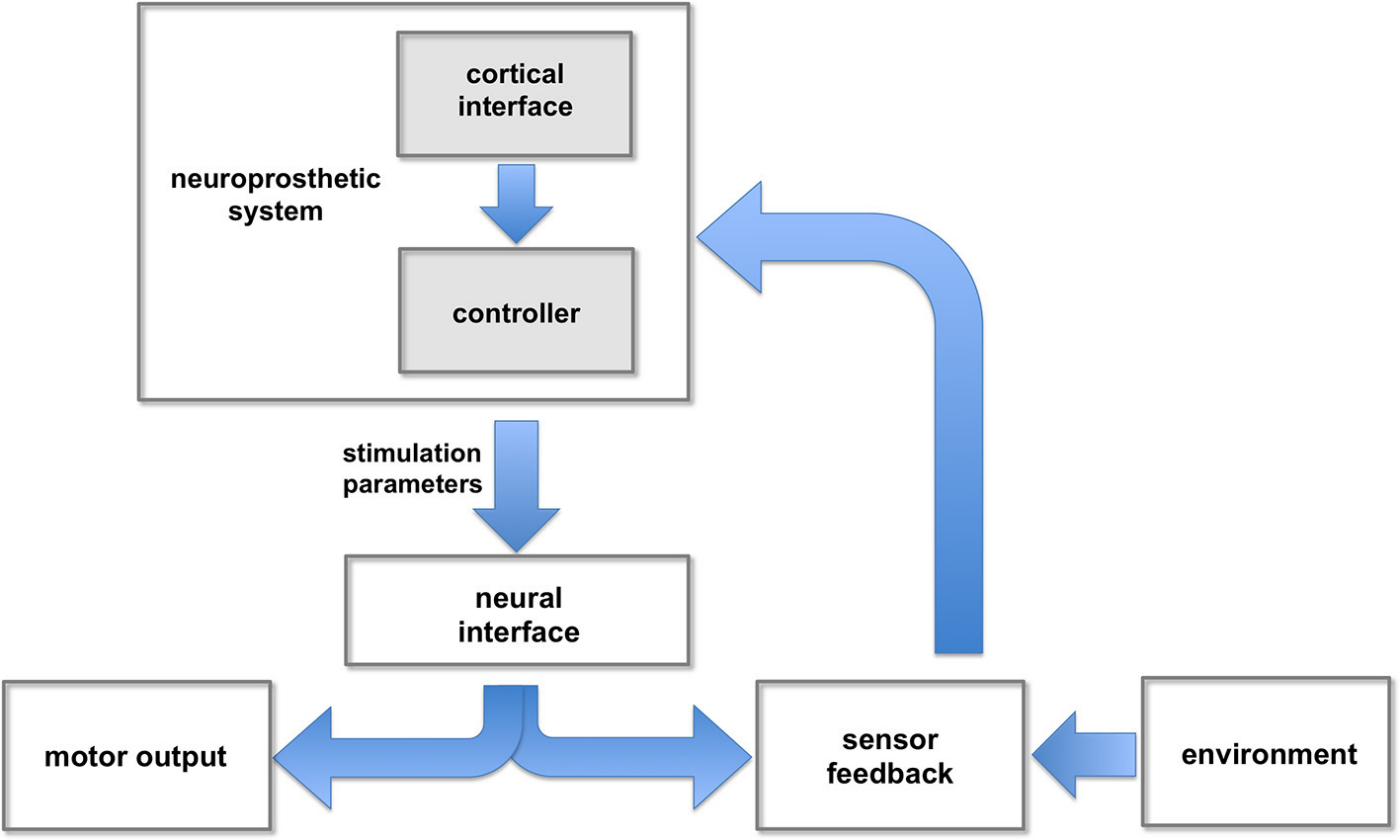
**Neuroprosthetic control**. The neuroprosthetic controller receives user commands (e.g., intended movement) extracted from cortical signals, and feedback information from different sensors. These inputs are combined and processed to adjust the stimulation parameters responsible for evoking intended movements.

**Figure 3:**
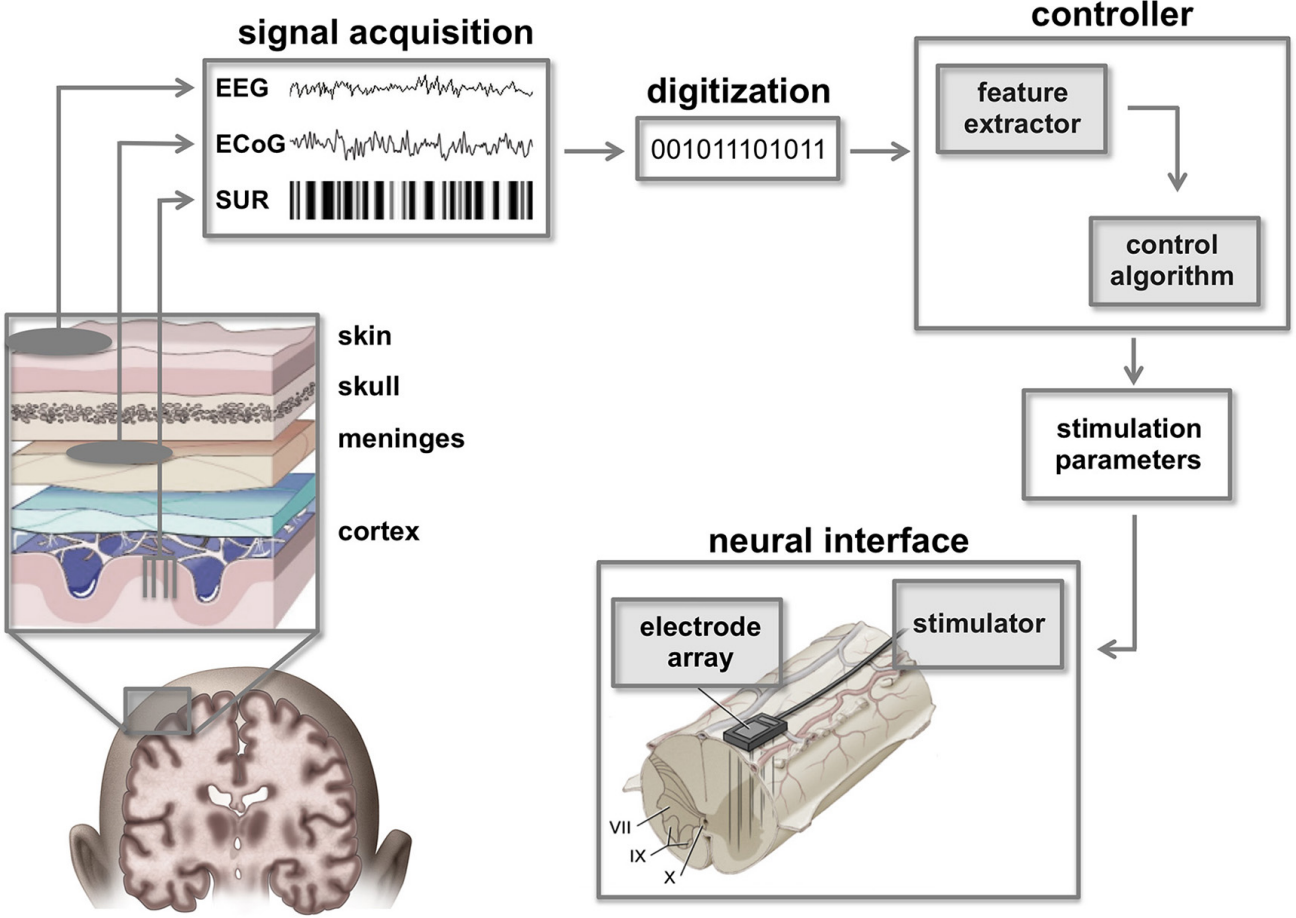
**Cortico-spinal neuroprostheses**. Command signals from the brain can be extracted using a variety of brain signal recording techniques such as single unit recordings (SUR), electrocorticographic signals (ECoG), or electroencephalographic signals (EEG). Raw signals must be digitized and filtered to extract essential features that can be classified by the controller in order to calculate appropriate stimulation parameters. In turn, these parameters are used by a neural interface to activate spinal circuitry below the level of injury. Figure adapted from Smart Draw LifeART Collection Images and Lobel and Lee (2014).

Brain machine interfaces (BMI) are neural interface systems that can record, analyze, and decode brain signals (Wang et al., 2010) to infer volitional intent, which in turn can be used to control limb movement and assistive devices (Figure 2) (Leuthardt et al., 2004; Hochberg et al., 2006; Schwartz et al., 2006; Miller et al., 2010; Carmena, 2012; Fifer et al., 2012). Brain commands may be recorded using sensors located on the scalp (electroencephalogram), the surface of the brain (electrocorticogram), or the brain parenchyma using intracortical electrodes that record activity from single neurons (single unit recording) or groups of neurons (local field potentials) (Figure 3). Electroencephalographic recordings offer a non-invasive recording technique that is safe and easy to implement. However, controlling multiple degrees of freedom with electroencephalographic signals has proven difficult due to challenges with extracting and classifying individual signal features as well as an inherent low spatial resolution (Yang et al., 2011). Single unit recordings and local field potentials offer excellent signal resolution, but are highly invasive (Buzsáki et al., 2012). Single unit recordings capture the activity of distinct neurons. The high spatial and temporal resolution provided by single unit recordings allows for precise measurements of neuronal spikes (Buzsáki et al., 2012). The downfall to single unit recordings is a difficulty isolating specific neural activity due to crosstalk from neighboring cells (Bai and Wise, 2001). Furthermore, single unit recordings can be biased toward activity from larger neurons adjacent to the intended neuron (Buzsáki et al., 1983). Finally, electrode migration, immune responses (e.g., glial scarring), and disruption of surrounding neural tissue interfere with signal quality and limit reliable single unit activity to acute recording conditions (Carter and Houk, 1993; Polikov et al., 2005). Local field potentials reflect a weighted average of integrative processes and associations between cells that can be detected over longer distances through extracellular space (Logothetis, 2003a,b; Andersen et al., 2004; Bronte-Stewart et al., 2009; Buzsáki et al., 2012; Rosa et al., 2012). Unfortunately, the longer recording range of local field potential techniques is associated with decreased spatial resolution. Electrocorticogram presents a good balance between risks and benefits, as it provides good spatiotemporal resolution without damaging underlying cortical tissue (Leuthardt et al., 2004; Wilson et al., 2006; Schalk et al., 2008; Moran, 2010; Slutzky et al., 2010).

Extracted brain signals must undergo filtering to remove movement artifacts and electrical noise before they can be used by a BMI and neuroprosthetic controller to generate motor commands (Kowalski et al., 2013). Filtered signals must be analyzed using classifiers and signal processing algorithms that identify unique features or signatures (Kowalski et al., 2013). In turn, these features are mapped to specific functions and/or degrees of freedom that control neuroprosthetic systems and assistive devices (Pfurtscheller et al., 2003; Musallam et al., 2004; Müller-Putz et al., 2005; Jackson et al., 2006; Moritz et al., 2008; Daly et al., 2009; Chadwick et al., 2011).

Pioneering work by Georgopoulos et al. used single unit recordings to establish a high degree of correlation between arm movement and cortical activity within a non-human primate (Georgopoulos et al., 1986). Subsequently, several studies in non-human primates and SCI-survivors have demonstrated stable, chronic, intracortical recordings using microelectrode arrays such as the Utah and Michigan arrays (Wessberg et al., 2000; Serruya et al., 2002; Taylor et al., 2002; Pfurtscheller et al., 2003; Suner et al., 2005; Cheung, 2007; Cheung et al., 2007; Moritz et al., 2008; Langhals and Kipke, 2009; Sharma et al., 2010, 2011; Do et al., 2011; Hochberg et al., 2012). Cortical signatures can be identified from their spatial, temporal, and frequency-dependent features (Nicolas-Alonso and Gomez-Gil, 2012). However, BMI application to complex neuroprosthetic control has been limited due to the difficulty of extracting sufficient numbers of unique signatures for control of systems with multiple degrees of freedom (Shih et al., 2012). Ongoing efforts in decoding algorithms, together with advances in neural training techniques such as motor imagery, have recently improved feature extraction, allowing SCI survivors to control complex movements using BMI (Wang et al., 2009, 2013; Chao et al., 2010; Yanagisawa et al., 2011).

## Conclusions

Recent advances in the fields of BMIs and electrical stimulation therapy provide a promising outlook for patients with SCI. However, it is clear that successful restoration of independence for SCI survivors requires integration of selective electrical stimulation techniques, feedback control, and optimal control algorithms. As is the case in normal human neurophysiology, selective muscle activation as well as integration of force feedback, balance, proprioception, and reduction of muscle fatigue are all critical for motor function. Therefore, next-generation closed-loop neuroprosthetic systems must integrate fully implantable multi-channel stimulators and feedback sensors with adaptive control systems. Furthermore, control algorithms must be designed for seamless integration with BMI systems and real-time processing, integration, and transmission of feedback control signals. Devices that are capable of coupling such novel stimulation, intention detection, proprioceptive sensing, and control algorithms are currently under development, with clinical translation just beyond the horizon. Ultimately, these technologies will provide SCI survivors with increased independence in daily life, improved overall health, and enhanced quality of life.

### Conflict of interest statement

Intellectual property licensed to Boston Scientific (J. Luis Lujan). The authors declare that the research was conducted in the absence of any commercial or financial relationships that could be construed as a potential conflict of interest.
